# Identification of progression markers for prostate cancer

**DOI:** 10.1080/15384101.2025.2563930

**Published:** 2025-09-29

**Authors:** Jie Song, Yang Zhou, Harald Hedman, Tommi Rantapero, Maréne Landström

**Affiliations:** a Department of Medical Biosciences, Pathology, Umeå University, Umeå, Sweden; b Genevia Technologies, Tampere, Finland

**Keywords:** AURKA/B, KIF23, TGFBR1, Cancer, prognostic modeling

## Abstract

TGFβ functions as a tumor suppressor or promoter, depending on the context, making TGFβ a useful predictive biomarker. Genes related to TGFβ signaling and Aurora kinase were tested for their ability to predict the progression risk of primary prostate tumors. Using data from The Cancer Genome Atlas (TCGA), we trained an elastic-net regularized Cox regression model including a minimal set of gene expression, copy number (CN), and clinical data. A multi-step feature selection and regularization scheme was applied to minimize the number of features while maintaining predictive power. An independent hold-out cohort was used to validate the model. Expanding from prostate cancer, predictive models were similarly trained on all other eligible cancer types in TCGA. *AURKA*, *AURKB*, and *KIF23* were predictive biomarkers of prostate cancer progression, and upregulation of these genes was associated with promotion of cell-cycle progression. Extending the analysis to other TCGA cancer types revealed a trend of increased predictive performance on validation data when clinical features were complemented with molecular features, with notable variation between cancer types and clinical endpoints. Our findings suggest that TGFβ signaling genes, prostate cancer related genes and Aurora kinases are strong candidates for patient-specific clinical predictions and could help guide personalized therapeutic decisions.

## Introduction

Cancer is the second leading cause of death worldwide, with an estimated 10 million people dying of cancer in 2020 [1]. Prostate cancer is the second most common cancer diagnosed in men worldwide and caused estimated 375,304 deaths in 2020 [2]. The American Cancer Society’s estimates that there will be approximately 299,010 new cases of prostate cancer and 35,250 deaths from prostate cancer in the United States for 2024 [3]. The incidence of prostate cancer detection has increased since introduction of the prostate-specific antigen (PSA) test in the 1990s. However, PSA test results in overtreatment, and does not affect mortality in prostate cancer [4]; thus, there remains a need for new prognostic biomarkers [5,6]. Currently, the grading system most commonly used for prostate cancer is the Gleason grading system, based on microscopic appearance [7]. Higher Gleason score (GS) indicates a poor prognosis. In 2014, a GS of 7 was separated into two different groups: GS 3 + 4 = 7 and GS 4 + 3 = 7, which differ in prognosis [8]. Although GS is a good clinical prognostic marker, there remains a need for robust molecular prognostic markers. In addition, urine liquid biopsies and potential serum biomarkers are also attractive and may play an important role in prostate cancer detection and the precision medicine revolution. For example, bone sialoprotein (BSP) and osteopontin (OPN) have been shown to be useful in the prognostication of prostate cancer. The higher the BSP level in prostate cancer patients, the shorter the time to bone metastasis. OPN can be used to assess the response of castration-resistant prostate cancer (CRPC) patients to chemotherapy [9]. Prostate development and homeostasis are dependent on male hormones, which signal through the androgen receptor (AR). This is instructive for treatment. In fact, abiraterone acetate plus prednisolone combined with androgen deprivation therapy (ADT) has a clinically important improvement for patients with prostate cancer in survival; enzalutamide and abiraterone should not be used concomitantly in men with prostate cancer who are starting long-term ADT [10]. Uncontrolled proliferation of prostate cancer cells is associated with dysregulated AR signaling, and *AR* amplification has been implicated as a reason for failure of ADT [11,12]. Additionally, *MYC* proto-oncogene, bHLH transcription factor (*Myc*) amplifications have been detected in 8% of prostate cancer cases [13].

Transforming growth factor β (TGFβ) signals via the cell membrane-bound serine/threonine kinase receptors, TβRI and TβRII. The TGFβ pathway regulates downstream targets and thereby plays key roles in both embryonic development and the maintenance of adult tissue homeostasis [14]. The effects of TGFβ depend on the cell type and cellular context. Key processes regulated by TGFβ include cell proliferation, apoptosis, differentiation, epithelial – mesenchymal transition, migration, invasion, angiogenesis, and immune responses. Dysregulation of the TGFβ pathway leads to many different diseases, including cancer and immune dysfunction [14–16]. Most carcinoma cells have inactivated their tumor-suppressor genes and benefit from tumor-promoter effects [16]. Based on the effects of the TGFβ pathway on tumor initiation and progression, many novel therapies have been developed, including small molecule inhibitors, antibodies, and TGFβ ligand traps [17,18].

Dysregulated cell proliferation is a hallmark of cancer [19]. Aurora kinases are a family of well-conserved serine/threonine kinases, including Aurora kinase A (AURKA), Aurora kinase B (AURKB), and Aurora kinase C (AURKC) [20]. They play key roles in the cell cycle – with AURKA and AURKB playing major roles in mitosis, and AURKC having a key role in meiosis [20]. They are involved in centrosome duplication and maturation, microtubule spindle formation, chromosome alignment and segregation, and cytokinesis [21,22]. AURKB is also an enzymatic component in the chromosomal passenger complex (CPC), which includes three other regulatory components: inner centromere protein (INCENP), survivin (encoded by *baculoviral IAP repeat containing 5, BIRC5*), and borealin (encoded by *kinesin family member 2C*, *KIF2C*) [23]. AURKB controls abscission timing by regulating the localization and function of vacuolar protein sorting 4 (VPS4) [24]. VPS4 is a core component of the endosomal sorting complex required for transport (ESCRT) machinery, functioning as an AAA-ATPase [25]. Moreover, AURKB phosphorylates kinesin family member 23 (KIF23) to regulate central spindle size and cytokinesis [26,27]. Since Aurora kinases play key roles in cell proliferation, they have become new therapy targets for cancer treatment, and several different inhibitors are currently evaluated in clinical trials [21,28,29].

We have previously shown that the endosomal APPL1 and APPL2, which are short for adaptor protein, phosphotyrosine interacting with PH domain and leucine zipper 1 and 2, are involved in the nuclear accumulation of the intracellular domain (ICD) of TβRI in response to TGFβ stimulation of cells [6,30]. APPL proteins promote the expressions of *AURKB*, *BIRC5*, and *cell division cycle associated 8* (*CDCA8*). Moreover, AURKB interacts with TβRI in the midbodies during mitosis. The AURKB-TβRI complex is a new biomarker for advanced prostate cancer [31]. TNF receptor associated factor 6 (TRAF6) is a E3 ligase, which can cause Lys63-linked polyubiquitination of APPL1 [32], TβRI [30] and AURKB [31]. Notably, *TRAF6* is amplified in 13.6% of neuroendocrine prostate cancer patients and 12.9% of CRPC patients [33,34]. Since TGFβ signaling is also a therapeutic target in cancer [15,18], there is great potential for use of a combination of TGFβ inhibitors and AURKB inhibitors in future therapeutic strategies.

In the present study, we assessed the prognostic significance of selected genes related to the TGFβ pathway, prostate cancer, and Aurora kinases, by applying machine learning methodology. We obtained publicly available molecular and clinical data about several different cancer types from The Cancer Genome Atlas (TCGA), and developed a machine learning framework utilizing elastic-net regularized Cox regression [35]. The genes included *AURKA, AURKB, KIF23, VPS4A, VPS4B, APPL1, APPL2, TGFBR1, phosphatase and tensin homolog (PTEN), MYC, tumour protein p53 (TP53), AR and TRAF6*. A new prognostic model was built and different genes and pathways associated with predictive power were identified. *AURKA, AURKB* and *KIF23* are the main genes which are involved in the prognostic model. A total of 109 prognostic models were trained to study 31 different cancer types, and 39 models could differentiate between high-risk and low-risk cancers when the prognostic models included molecular and clinical features. Of these 39 models, 24 models performed better than models without molecular features.

## Materials and methods

### Cell culture

Human prostate cancer cell line PC-3 U was cultured in RPMI-1640 medium supplemented with 10% fetal bovine serum (FBS), 2 mM L-glutamine, 100 U/ml penicillin, and 0.1 mg/ml streptomycin, as previously described [6,31]. Nocodazole (400 ng/ml) was purchased from Sigma.

### Antibodies used for immunoblotting

Antibodies against the following proteins were used for immunoblotting: APPL1 (Cell Signaling Technology Cat# 3858), APPL2 (Santa Cruz Biotechnology Cat# sc-67403, RRID:AB_2056383), VPS4A (Abcam Cat #ab229806), VPS4B (Abcam Cat #ab137027), β-tubulin (Cell Signaling Technology Cat# 2146, RRID:AB_2210545), and Horseradish peroxidase – coupled secondary antibodies were purchased from Dako and Protein-G Sepharose and ECL immunoblotting detection reagents from GE Healthcare. PageRuler Prestained Protein Ladder was from Thermo Fisher Scientific.

### Protein analysis

Cells were washed twice with ice-cold phosphate-buffered saline (PBS) and lysed in ice-cold lysis buffer [150 mM NaCl, 50 mM Tris (pH 8.0), 0.5% (v/v) sodium deoxycholate, 1% (v/v) NP-40, 10% (v/v) glycerol, supplemented with protease inhibitors]. Following lysis, the samples were centrifuged, and the supernatant was collected. Total protein concentration was quantified using the BCA protein assay (Thermo Fisher Scientific). Equal amounts of protein lysates were resolved by SDS-polyacrylamide gel electrophoresis (SDS-PAGE) on Mini-PROTEAN® TGX™ gels (Bio-Rad) and subsequently transferred to nitrocellulose membranes.

### siRNA transfection

On TARGET plus *APPL1* (5′-GAUCUGAGUCUACAAAUUU-3′), *APPL2* (5′-AGAUCUACCUGACCGACAA-3′;), and siGENOME non-targeting control siRNA (target sequence, 5′-UAGCGACUAAACACAUCAA-3’) were obtained from Dharmacon Research. siRNA was transfected into cells using Oligofectamine Transfection Reagent (Thermo Fisher Scientific), according to the manufacturer’s protocol.

### Data retrieval

In this study, public molecular and clinical data were used from TCGA. We downloaded RNA-seq expression data, copy number (CN) data, and clinical metadata from the harmonized Genomic Data Commons (GDC) database release 24 [36] using the R v.4.1.3 [37] package TCGAbiolinks v. 2.16.4 [38].

### Data preprocessing

For the RNA-seq normalized count data, variance stabilizing transformation (VST) was applied using the *vst* function from R package DESeq2, v. 1.38.2 [39], with the *blind* parameter set as true. In order to avoid leakage of information from the training data to the validation set the *VST* was applied separately for the training and validation datasets. The already preprocessed CN data were utilized as is. Only samples originating from primary tumors were retained in the analysis. In the rare cases where two or more primary tumor samples were available for the same patient, only one representative sample were selected, to avoid selecting multiple samples from the same patient.

### Univariate survival analysis for prostate and breast adenocarcinoma

Univariate survival analysis was performed for the prostate adenocarcinoma (PRAD) preprocessed datasets by including only individuals for whom both expression and CN variant data were available. In the analysis, expression and CN status of 13 genes (Supplementary Data 1), and two clinical features (age at diagnosis and Gleason group) were tested for association with four distinct clinical endpoints: overall survival (OS), disease-specific survival (DSS), progression-free interval (PFI), and disease-free interval (DFI) (endpoints defined in Supplementary Data 2). For the gene expression-based features, analysis was performed by categorizing the individuals into two groups based on the median expression. CN features were evaluated by assigning the individuals into CN neutral and mutated groups, by treating CN deletion and amplification as distinct features. To compare the survival outcomes of the two groups, log-rank tests were performed and Kaplan-Meier curves were plotted using *surv_fit* and *ggsurvplot* functions, respectively, from the R package survminer v. 0.4.9 [40]. The univariate analysis was performed similarly to breast adenocarcinoma with the exception of selecting age at diagnosis and tumor stage as clinical features.

### Studying the associations of molecular features with Gleason group and tumour stage

To test the association between each expression feature and the Gleason group, a t-test was performed using the R-function *t_test* from the package rstatix [41]. Each CN feature was tested by comparing the proportions of alteration carriers and non-carriers between the Gleason groups, using Fisher’s exact test. The test was performed using the R-function *pairwise_fisher_test* from the package rstatix. To evaluate the association between expression features and tumor stage, the Pearson correlation was calculated using the *cor.test* function from the stats R package. The results were visualized using box plots and bar plots, prepared using the *ggboxplot* and *ggbarplot* functions, respectively, from the package ggpubr [42].

### Prognostic model development

Similarly to the univariate analysis, the prognostic model development and evaluation were performed using the pre-processed datasets, including only individuals for whom both expression and CN variant data were available. Feature selection was applied for this initial set, utilizing a two-step procedure. During the first step, patients were split into training and validation sets, using a 75/25 split. Features were then pre-filtered based on the following criteria. An expression-based feature was filtered out if its median expression in the training set was ≤ 20, or if the percentage of individuals in the training set expressing that feature was ≤ 25%. CN-based features were filtered out if ≤ 15% of individuals carried neither deletion nor amplification.

During the feature selection step, the remaining features were tested for association with each of the four clinical endpoints, similarly to in univariate analysis. However, instead of using the full dataset, only the training set was included. Features found to be significantly associated with the clinical endpoint (log-rank p-value < 0.05) were selected for further prognostic model development. The remaining features were combined with a set of clinical features, including age at diagnosis, gender, tumor stage, and Gleason group (full descriptions provided in Supplementary Data 3).

Tumor stage was treated as a dummy variable, such that for each cancer type, the lowest observed stage was treated as the baseline, and each observed higher stage was compared to the baseline. The elastic-net regularized Cox regression model was then trained using the R package glmnet v.1.4.8 [43]. Lambda parameter optimization for the models was performed using the *cv.glmnet* function, with the number of iterations set to 100,000, and Harrel’s concordance as the error metric. After optimization, the final model was fit using the *glmnet* function.

**Figure 1.**
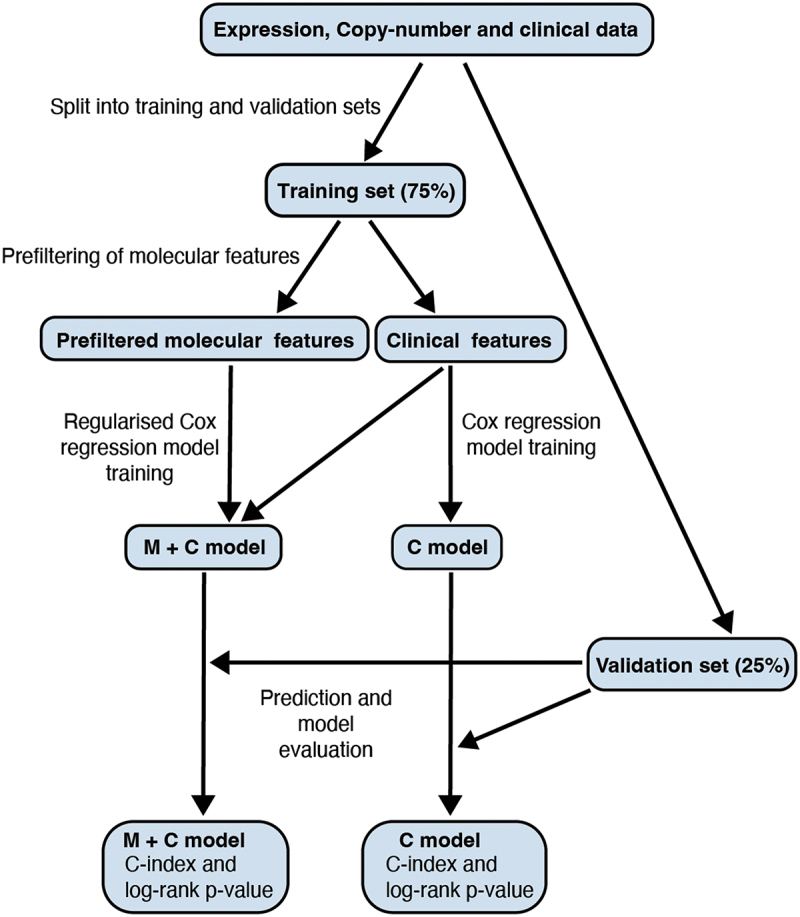
Schematic of the workflow of prognostic model development. M + C model: model including both molecular and clinical features; C model: model including clinical features.

The trained penalized Cox regression models were used to predict patient-specific risk scores for individuals in the validation set. Predictions were obtained using the *predict* function from the R package stats. Then the model’s performance was evaluated by splitting the validation cohort into two groups based on the median predicted risk score. Kaplan-Meier curves were fitted, and the log-rank test was performed to assess whether the two groups exhibited different survival outcomes. Furthermore, C-indices were calculated using the *concordance.index* function from the R package survcomp v. 1.48.0 [44]. Finally, the performances of the developed models were compared against models that had been trained using only the clinical features. For these models, the training was performed using standard Cox regression using the *coxph* function in the R package survival v. 3.5.7 [45]. Training and evaluation were performed using the sets that were used for the prognostic models developed including the molecular features. The prognostic model development is illustrated in Figure 1.

### Differential expression (DE) and pathway enrichment analysis

DE analysis was conducted using the DESeq2 functions *DESeq* and *results*, with the absolute log2 fold change threshold set to 0.5, and with the parameter *lfcThreshold* as the latter function. Genes were considered differentially expressed if the adjusted p-value (Benjamini-Hochberg) was < 0.05. Pathway enrichment analysis was performed as overrepresentation analysis using the R package clusterProfiler v.4.2.2 [46] and ReactomePA v.1.42.0 [47] against the Reactome database. Pathways were considered significantly enriched if the adjusted p-value (Benjamini-Hochberg) was < 0.05.

### Extension of the PRAD prognostic model for PFI

The top 300 differentially expressed genes from the DE analysis between high and low risk PFI groups were selected for improving the PFI prognostic model for PRAD. To extend the model we first reconstructed the original prognostic model such that original features were used to fit a non-regularized cox-regression model based on the training data. Then the top 300 DE features were added one by one at the time to the model in the order dictated by the Kaplan-Meier log-rank p-values, which obtained for the features based on the analysis on the training set. We observed the model performance during this stepwise model extension procedure and in the end selected the model with the lowest log-rank p-value.

As an alternative approach, we first applied a feature-elimination step to pre-filter and order the DE gene-based features. The feature elimination was performed by training a random survival forest model (RSF) using the training set from which the variable importances were extracted. Based on the variable importance the 20% of the features with the lowest variable importance were filtered out. This process was continued until less than 10 features was remaining. During the elimination process, the model performance was measured using the Out of Bag Error (OOB) and the feature set which produced the lowest OOB was selected for the stepwise addition phase which was performed similarly as described above. In the feature elimination the hyper parameters were tuned and the RSF model was fit using the tune and rfsrc functions respectively and the variable importances were obtained using the vimp function in R-package randomForestSRC v. 3.2.2 [48].

In order to assess whether a more versatile selection of genes would improve the current model, a list of known oncogenes was downloaded from oncoKB (https://www.oncokb.org/) [49]. Furthermore, since our original gene list involves genes associated with the cell cycle and TGFβ pathways, we extended the list of oncogenes with genes from these pathways. The genes lists were collected from QuickGO [50] (https://www.ebi.ac.uk/QuickGO) based on gene ontology terms “cell cycle” and “transforming growth factor beta receptor signaling pathway.” The TGFβ gene list was further extended by selecting genes based on literature. The full list of genes is presented in Supplementary Data 4. We applied the KM analysis-based ranking and the feature elimination procedure followed by the stepwise model extension as described as above.

## Results

### Univariate analysis of gene-based and clinical features in PRAD

**Figure 2.**
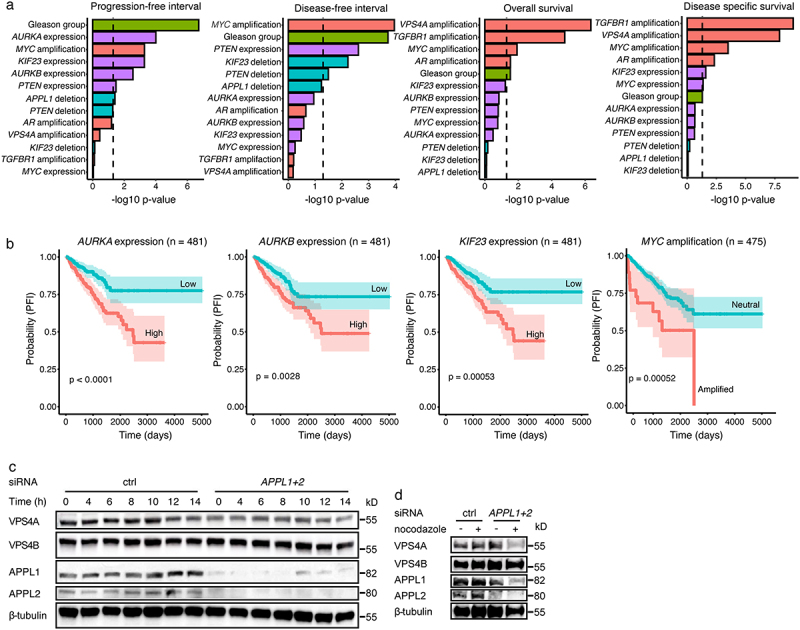
Results from the univariate survival analysis in PRAD. (a) Survival analysis log-rank test results. Bar plot shows the −log10 p-values obtained for molecular and clinical features, based on univariate analysis including the full PRAD cohort. Only features found to be significantly associated with at least one clinical endpoint are shown. Dashed line indicates the threshold for significant features. (b) Kaplan-Meier curves for the top four predictive molecular features for progression-free interval (PFI) based on univariate analysis including the full PRAD cohort. (c) PC-3 U cells were synchronized by double thymidine block and treated with or without *APPL1* and *APPL2* siRNA. Cells were released into fresh growth medium and cell lysates were prepared at different times for immunoblot analysis. (d) PC-3 U cells were transfected with or without *APPL1* and *APPL2* siRNA, incubated with nocodazole for 12 h, and then immunoblot analysis was performed.

Kaplan-Meier analysis revealed that several gene-based features were associated with survival in PRAD, including *TGFβRI* and *VPS4A*. However, due to the low numbers of events for OS (*n* = 10) and DSS (*n* = 5), these results must be interpreted cautiously. Unsurprisingly, the same top features were associated with OS and DSS, while different top features were associated with PFI and DFI (Figure 2(a)). High expressions of *AURKA*, *AURKB*, and *KIF23* were found to be associated with shorter PFI (Figure 2(b)). *MYC* amplification was associated with both shorter PFI and DFI (Figure 2(b), Supplementary Figure S1(a)). Interestingly, *VPS4A* and *VPS4B* showed different effects on survival outcomes: *VPS4A* amplification was associated with OS and DSS, while *VPS4B* did not show such an association. Given that AURKB are downstream targets of APPL1 and APPL2 [31], we speculated that APPL may also regulate the expression of VPS4A and thus, we used siRNA to reduce APPL1 and APPL2 expression in human prostate cancer PC-3 U cells. Notably, we found that VPS4A expression was significantly reduced, while VPS4B was not affected (Figure 2(c, d)), which may explain the selective association of *VPS4A* with OS and DSS. Furthermore, low *PTEN* expression was associated with shorter PFI and DFI (Supplementary Figure S1(b, c)). Additionally, CN deletion of *PTEN* was associated with shorter DFI, and high *TGFB1* expression was associated with shorter PFI (Supplementary Figure S1(d, e)).

### Development of a prognostic model for PRAD

**Figure 3.**
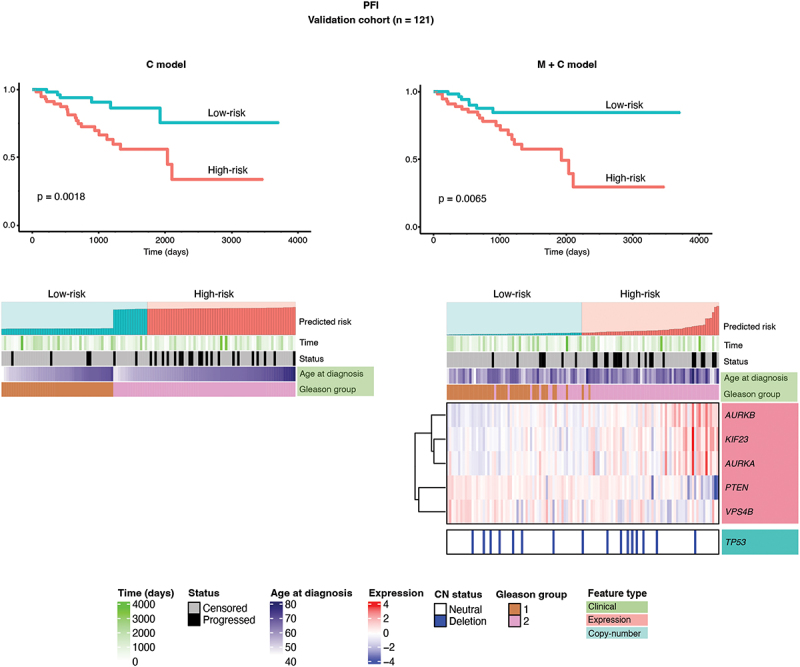
Illustration of prognostic models obtained for PRAD. Kaplan-Meier curves for high-risk and low-risk groups predicted by the prognostic models for PFI, and associated heat-maps. The survival probabilities for high-risk and low-risk groups are shown by the red and blue curves, respectively. The heat-maps illustrate the features included in the prognostic models. Gleason group 1: GS ≤ 6 or GS = 3 + 4; Gleason group 2: GS = 4 + 3 or GS ≥ 8. Expression values have been scaled using z-transformation.

The model training yielded models including both molecular and clinical features (M + C models), and corresponding models without molecular features (C models), for PFI, DFI, and DSS. We could not evaluate a prognostic model for OS due to the lack of events in the evaluation set for OS. The log-rank test revealed the significance of the M + C model for PFI (*p* = 0.0065). Comparing the C-indices between the prognostic models showed that the M + C model for PFI exhibited a modest improvement over the C model for PFI. The evaluation results are summarized in Table 1 and Figure 3. Notably, 6 of 13 genes (*AURKA, AURKB, KIF23, PTEN, VPS4B*, and CN status of *TP53*) were included in the prognostic model for PFI (Figure 3).

**Table 1.** Evaluation results for PRAD prognostic models.

	C model			M + C model		
Clinical endpoint	Log-rank p-value	C-index	C-index conf. interval	Log-rank p-value	C-index	C-index conf. interval
PFI	0.0018	0.6253	(0.3855–0.8161)	0.0065	0.6276	(0.3876–0.8177)
DFI	0.062	0.5802	(0.2488–0.8523)	0.300	0.5522	(0.2297–0.8361)
DSS	0.45	0.7941	(0.0438–0.9969)	0.3711	0.7647	(0.0406–0.996)

PFI, progression-free interval; DFI, disease-free interval; DSS, disease-specific survival.

Furthermore, to gather a comprehensive list of genes which could be associated with poor survival, a list of known TGFβ and cell cycle pathway genes was retrieved as well as known oncoKB (Supplementary Data 4) [49,50]. Two alternative gene ranking strategies were performed for both of these gene lists for a model extension procedure in which one gene feature was added at a time and observed the performance of the new model for the evaluation data. As a result, no significant improvements was observed (Supplementary Results Table S1).

### Association of molecular features with Gleason group

Including molecular features moderately improved the prognostic power of PFI in PRAD; however, the difference was not significant. It was next investigated whether this was due to the fact that the molecular features correlate with the Gleason group. Considering the full PRAD cohort, the association between each expression feature and Gleason group was tested by comparing the mean expressions of each gene in the two groups (Gleason group 1: GS ≤ 6 or GS = 3 + 4; Gleason group 2: GS = 4 + 3 or GS ≥ 8). Similarly, each CN feature was tested by comparing the proportions of CN-alteration carriers and non-carriers between the two Gleason groups. The results confirmed that the expressions of genes *AURKA, AURKB, KIF23, PTEN, VPS4B* and *TP53* which were included as features in the PFI prognostic model were all associated with the Gleason group (Supplementary Figure S2(a)). The expression of other genes of interest, such as *AR* and *VPS4A*, was also associated with the Gleason group (Supplementary Figure S2(a)). Additionally, we found that the proportion of *PTEN*-deletion carriers was higher in Gleason group 2 (Supplementary Figure S2(b)). These findings support that the Gleason group already carries a major proportion of the prognostic power of these genes and vice versa.

### DE Analysis between predicted high-risk and low-risk groups for PFI in PRAD

**Figure 4.**
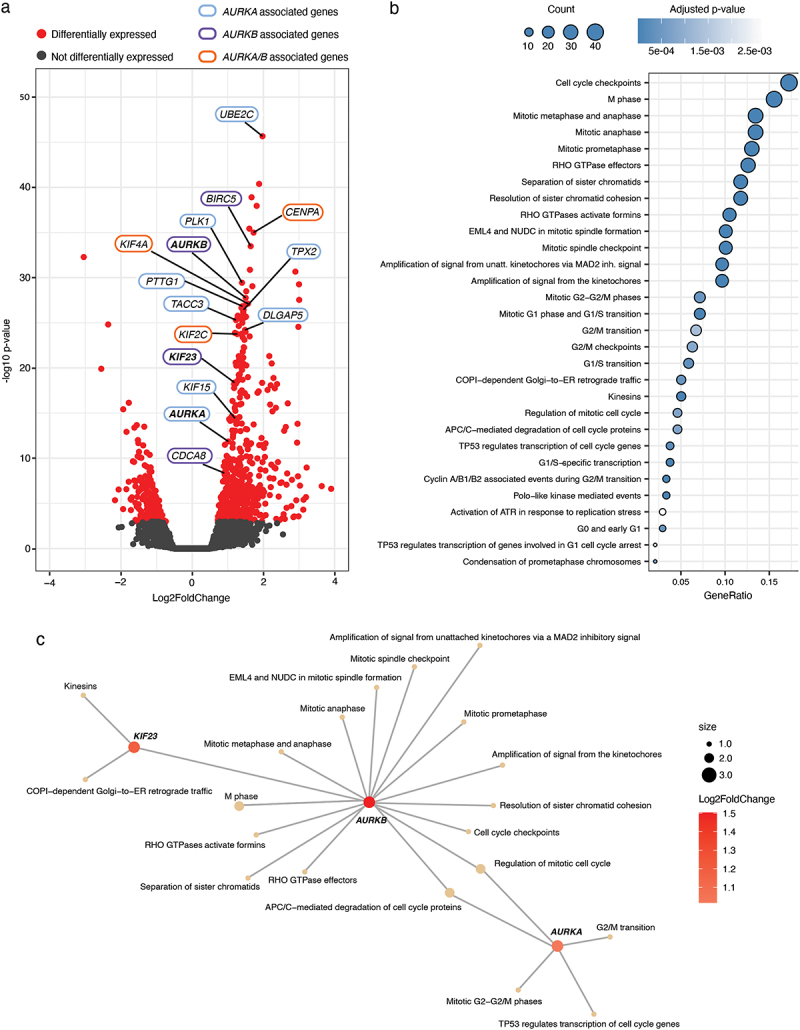
Results from transcriptomic analysis based on high-risk and low-risk PFI groups. (a) Volcano plot illustrating the differentially expressed genes (Absolute log2FoldChange > 0.5, adjusted *p* < 0.05) between high-risk and low-risk groups for PFI. Y-axis corresponds to −log10 transformed p-value, and x-axis corresponds to the Log2FoldChange. Differentially expressed genes are highlighted in red. Selected *AURKA*-related genes are highlighted with blue, whereas *AURKB*-related genes are highlighted in purple. Genes related to both *AURKA* and *AURKB* are highlighted in orange. (b) Dot plot showing the top 30 enriched pathways from Reactome overrepresentation analysis performed based on differentially expressed genes for PFI. The colour of the circles represents the adjusted p-value (Benjamini-Hochberg), and the count represents the total number of observed differentially expressed genes associated with the pathways. The x-axis corresponds to the proportion of observed differentially expressed genes among the total number of genes in the pathway. unatt.: unattached; inh.: inhibitory. (c) Network plot illustrating the relationship between *AURKA, AURKB, KIF23* and the top 30 enriched pathways. Each node (circle) represents either a gene or a pathway. The edges (connections between the nodes) represent associations between genes with pathways. The pathway node size represents the number of genes associated with the pathway, and the intensity of the node color represents the observed Log2FoldChange between high-risk and low-risk PFI groups.

To better understand the underlying biological pathways associated with the predictive power of the prognostic model for PFI in PRAD, we performed differential gene expression analysis between the predicted high-risk and low-risk groups, followed by pathway enrichment analysis. We identified a total of 785 DE genes (adjusted *p* < 0.05), of which 587 were downregulated and 198 were upregulated (Figure 4(a), Supplementary Results Table S2). Many *AURKA* upstream and downstream genes were identified, including *UBE2C* [51], *TPX2* [52,53], *PTTG1* [54], *PLK1* [55], *DLGAP5* [56], *KIF15* [57], and *TACC3* [58], indicating the dysregulation of AURKA-related pathways in the cancers. We also identified many *AURKB*-related genes (such as *BIRC5*, *CDCA8*, and *KIF23*), as well as *KIF2C* [59,60], *CENPA* [61,62] and *KIF4A* [63,64], which are common substrates of AURKA and AURKB. Interestingly, the high expressions of *BIRC5*, *CDCA8*, and *KIF2C* were found to be associated with shorter PFI (Supplementary Figure S3 (a – c)). Based on overrepresentation analysis including the DE genes, we found that the top 30 enriched pathways were mainly related to cell cycle (Figure 4(b), Supplementary Results Table S3). Further inspection revealed that 3 of the 6 genes included in the prognostic model (*AURKA, AURKB, KIF23*) were heavily involved with the majority of these pathways (Figure 4(c)).

### Prognostic model development for TCGA cancer types

We trained a total of 109 prognostic models, covering 31 distinct cancer types. Prognostic models could not be generated for every cancer type and clinical endpoint, due to a lack of molecular data, significant features after the feature selection, or sufficient number of events associated with a clinical endpoint. Notably, a prognostic model was developed for the combination of colorectal adenocarcinoma (COAD) and rectal adenocarcinoma (READ), which were generally considered as a single cancer type (colorectal cancer). The training and validation set for each combination of cancer type and clinical end point have summarized in Supplementary Data 5 including statistics of the clinical features used in the model training.

**Figure 5.**
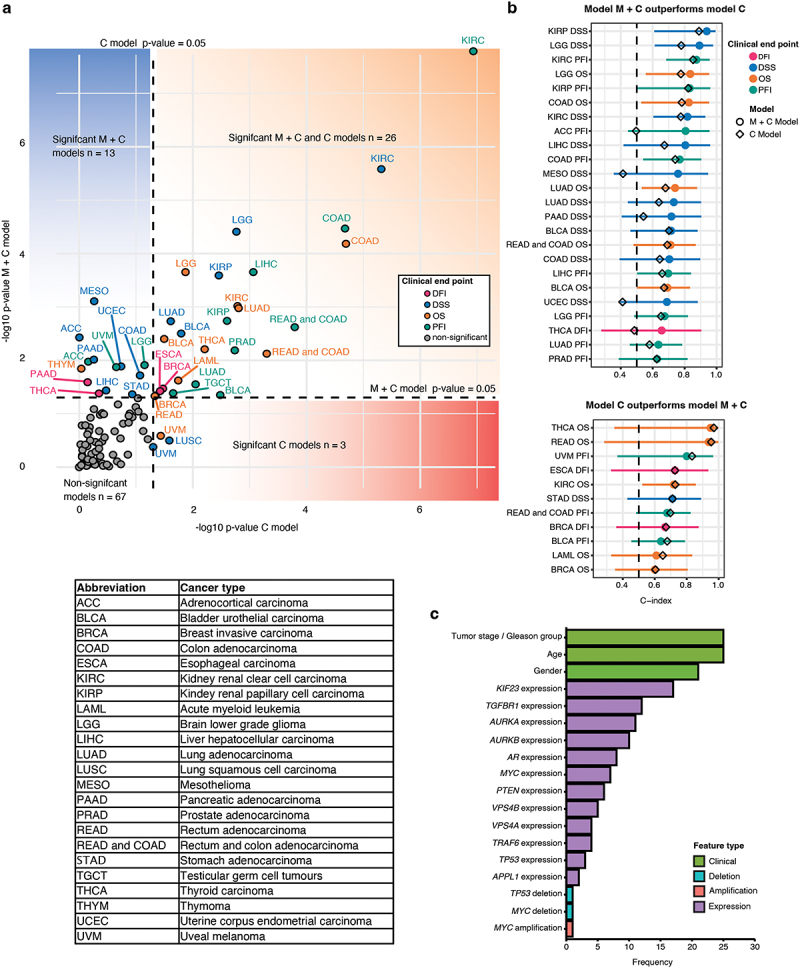
Evaluation results for the prognostic models for TCGA cancer types. (a) Scatter plot summarizing the evaluations results for all the trained models (*n* = 109). Each point represents a model trained for a cancer type and clinical endpoint. The x-axis represents the −log10 transformed p-value obtained for the model that has been trained excluding the molecular features. The y-axis represents the −log10 transformed p-value obtained for the model that has been trained including the molecular features. The color of the points illustrates the clinical endpoint, and the label associated with the point represents the cancer type. Dashed lines illustrate the thresholds for significant models. Table describes the abbreviations shown in the panel. (b) Forest plot comparing the performance of the significant models (log-rank *p* < 0.05, *n* = 35) between the prognostic models trained with inclusion of molecular features (circles) or exclusion of molecular features (diamond shapes). The color of the circle illustrates the clinical endpoint of the model. (c) Bar plot showing the frequencies of included features in the significant models.

Our evaluation revealed that a total of 39 M + C models were able to differentiate between high-risk and low-risk individuals (*p* < 0.05) according to the log-rank test. These models covered a total of 22 distinct cancer types. The corresponding total number of significant C models was 29. Further inspection revealed that both the M + C model and C model were significant for 26 combinations of cancer types and clinical endpoints. Moreover, for 13 combinations, a significant model was achieved only when including the molecular features. These results indicated that molecular features were quite powerful in these cancer types. Surprisingly, for 3 combinations, a significant model was achieved only when excluding the molecular features. The evaluation results, according to the log-rank test, are summarized in Figure 5(a).

To evaluate the performance of the significant M + C models against the corresponding C models, we compared the C-indices of the models. Among the 39 significant M + C models, 24 outperformed the corresponding C model, indicating that these molecular features may play a role in the prognosis of these cancer types. In 11 instances, the C model outperformed the corresponding M + C model, suggesting that other molecular features may be needed. The C-index could not be determined for 4 combination of cancer type and clinical endpoint. The evaluation results are summarized in Figure 5(b), and the full evaluation results are presented in Supplementary Results Table S4. When investigating the frequencies of features selected in the 39 significant models, we found that *AURKA*, *AURKB*, *KIF23 and TGFBR1* were among the most frequently included features, along with the clinical features (Figure 5(c)). Interestingly, *TGFBR1* amplification plays an important role in PFI, DFI, OS and DSS in BRCA (Supplementary Figure S4). This suggests that these genes play important roles in tumor development across many cancer types.

### Association of molecular features with tumour stage

**Figure 6.**
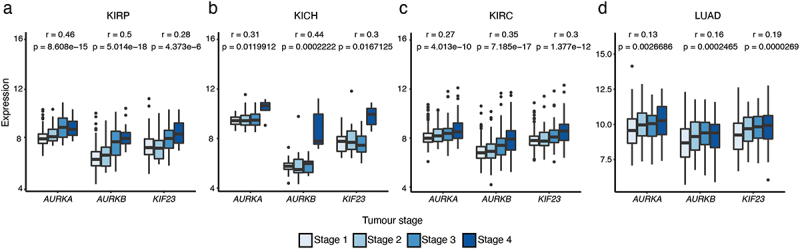
The relationship between top molecular features and tumor stage. (a – d) Box plots showing the distribution of expressions of *AURKA*, *AURKB*, and *KIF23* for the four groups corresponding to the four cancer stages in kidney renal papillary cell carcinoma (KIRP), kidney chromophobe (KICH), kidney renal clear cell carcinoma (KIRC), and lung adenocarcinoma (LUAD). Pearson correlations and p-values are shown.

Since a clear association was observed between the Gleason group and the molecular features included in the trained prognostic model for PRAD, we next studied whether a similar association was observed between these molecular features and the tumor stage in other cancer types. Similar to the associations with Gleason group, elevated expressions of *AURKA, AURKB*, and *KIF23* were associated with higher tumor stage in several cancer types, including kidney renal papillary cell carcinoma (KIRP), kidney chromophobe (KICH), kidney renal clear cell carcinoma (KIRC), and lung adenocarcinoma (LUAD) (Figure 6(a-d)).

In bladder urothelial adenocarcinoma (BLCA), KIRP, READ, and stomach adenocarcinoma (STAD), we found that increased *TGFBR1* expression was associated with tumor stage (Supplementary Figure S5(a)). Moreover, increased *MYC* expression was associated with tumor stage in KIRP, pancreatic adenocarcinoma (PAAD), and testicular germ cell tumors (TGCT) (Supplementary Figure S5(b)). Interestingly, we observed a similar trend of association between *TP53* expression and tumor stage in cholangiocarcinoma (CHOL), KIRP, and TGCT (Supplementary Figure S5(c)). In many cases the molecular features that were associated with tumor stage were also included in prognostic models for the cancer type in which the association was evaluated (Supplementary Results Table S5). This suggests that similar conclusions can be made regarding the relationship between prognostic molecular features and tumor stage, as with the Gleason group. Overall, these findings further strengthen support for the roles of genes related to the TGFβ pathway and Aurora kinases in tumor development of various cancer types.

## Discussion

TGFβ signaling pathways play a double-edged sword role in cancer. TGFβ acts as a tumor suppressor in normal and premalignant cells by regulating proliferation, differentiation and so on. But TGFβ can also act as a tumor promoter by evading immune surveillance and inducing tumor growth, migration and invasion [65]. It was observed that *TGFBR1* amplification in PRAD was associated with poor prognosis but due to the low number of observations in the investigated cohorts no clear conclusion about its causality could be drawn and we will therefore follow up on this in future studies (Figure 2(a)). Breast cancer (BC) can be divided into three categories based on molecular and histological evidence: BC expressing hormone receptor (estrogen receptor (ER+) or progesterone receptor (PR+)), BC expressing human epidermal receptor 2 (HER2+) and triple negative breast cancer (TNBC) (ER−, PR−, HER2−) [66]. ER, PR, HER2, and Ki67 serve as prognostic tests for breast cancer patients [66]. *TGFBR1* amplification was found to correlated to poor prognosis in BRCA (Supplementary Figure S4). Moreover, *TGFBR1* was identified as one of the most frequent molecular features in the 39 significant models (Figure 5(c)). An increased expression of *TGFBR1* in BLCA, KIRP, READ and STAD was found to be significantly associated to increased tumor stage and thereby poor prognosis (Supplementary Figure S5(a)). We have recently reported that the TβR1 protein forms a functional complex with AURKB which is required for proliferation of CRPC cells, and that this complex could be a useful biomarker for identification of aggressive prostate cancer [31]. Moreover, silencing of *TGFBR1* in a preclinical metastatic castration-resistant cell-line (PC-3 U) resulted in reduced growth of the orthotopic primary tumor and metastasis to regional lymph nodes [67]. All these results indicate the vital role of *TGFBR1* and its protein play an important role in tumor progression and tumor prognosis.

Mitosis – the process by which the duplicated genome is precisely separated into two identical sets of chromosomes by the microtubule spindle apparatus – is a highly controlled biological process [68]. Normal cells and tissues carefully control proliferation through the regulation of cell growth and division cycles. Cancer cells have developed different mechanisms to circumvent normal regulation of homeostasis, including cell cycle checkpoints, leading to uncontrolled and sustained proliferation [19]. Loss of cell cycle checkpoint control induces genetic instability. Based on the important role of the cell cycle in cancer development [69], it is not surprising to find that the major enriched pathways in our DE analysis were related to the cell cycle (Figure 4(b), Supplementary Results Table S3).

Members of Aurora kinase family have been identified as key mitotic and meiotic regulators that are required for chromosomal stability [20]. Aurora kinases are frequently overexpressed in many different cancers [29,70], including KIRP, KIPC, LUAD, BRCA [71], non-small cell lung cancer (NSCLC) [72], and prostate cancer [73]. Interestingly, AURKB expression is a predictor of reduced OS in NSCLC [74], glioblastoma [75], and gastric cancer [76]. Furthermore, the expressions of AURKA and AURKB both reportedly correlate with a poor OS in KIRC, KIRP, and LUAD [71]. In lung cancer, high KIF23 expression is observed and associated with worse OS, and inhibition of KIF23 expression leads to suppression of lung cancer cell growth [77]. In this study, it was found that high expressions of *AURKA*, *AURKB*, and *KIF23* were associated with shorter PFI in prostate cancer patients (Figure 2). MYC can promote the expressions of both AURKA and AURKB [78,79], which may be one reason why *MYC* amplification was also correlated with a poor PFI (Figure 2(b)). *AURKA*, *AURKB*, and *KIF23* were identified in our prognostic model for PFI in PRAD (Figure 4(a)) and were also involved in the top 30 enriched pathways (Figure 4(b)), indicating that these three genes may be a good prognosis biomarker in PRAD. Moreover, *AURKA*, *AURKB*, *KIF23* and *TGFBR1* were identified as the most frequent molecular features (Figure 5(c)), and their expressions were correlated with higher tumor stage in many different cancer types (Figure 6, Supplementary Figure S5(a)). These results demonstrate the correlation between the expression levels of *AURKA, AURKB, KIF23* and *TGFBR1* and tumor prognosis.

The distinct prognostic associations of VPS4A and VPS4B observed in our study suggest that these closely related genes may play divergent roles in prostate cancer progression (Figure 2(a)). Notably, we found that APPL proteins selectively regulate VPS4A but not VPS4B (Figure 2(c, d)), supporting the notion that VPS4A is integrated into key oncogenic pathways. Moreover, the expression of APPL1 is also increased in the prostate cancer [80]. This is consistent with emerging evidence that endosomal machinery, particularly the ESCRT system, contributes to cancer cell survival and metastasis [25,81].

The Gleason grading system is a powerful tool for predicting prostate cancer prognosis using samples from biopsies or radical prostatectomies. It is interesting to find that the expressions of *AR*, *AURKA*, *AURKB*, *KIF23*, *PTEN*, *TP53*, *VPS4A*, and *VPS4B* are related to GS (Supplementary Figure S2), indicating their significant roles in cancer development and prognosis. Furthermore, the high correlation between GS and genes of interest may be the reason why molecular features did not significantly improve the PRAD prognostic model (Table 1).

In this study, we used a defined set of molecular features (gene expression), and clinical features, to generate a total of 109 prognostic models, which were trained to study 31 different cancer types. When the prognostic models included both molecular and clinical features (M + C model), 39 models were able to distinguish between high-risk and low-risk cancers. Among these 39 models, 24 M + C models exhibited better performance than the corresponding C models (Figure 5(a, b)). In other words, the developed prognostic models showed improved performance when molecular features were considered. In an ideal setting, introducing more features during the model training should not lead to poorer performance. These results suggest that the methodology behind the model development must be improved to better select the most optimal set of features. In future studies, alternative machine learning methodology could be considered, such as random survival forests [82].

## Supplementary Material

Supplemental Material

Supplementary data 5.xls

Supplementary data 4.xlsx

Supplementary results table 3.xlsx

Supplementary results table 2.xlsx

Supplementary data 2.xlsx

Supplementary results table 5.xlsx

Supplementary results table 1.xlsx

Supplementary results table 4.xlsx

Supplementary data 3.xlsx

Supplementary data 1.xlsx

## Data Availability

Datasets and additional documents generated, analyzed, or used during the current study are available at https://github.com/MLandstromLab/publication_code/tree/main/CellCycle
