# Supplementary material for: Identification of progression markers for prostate cancer
Source: Cell Cycle. 2025 Sep 29;24(17-20):382–99. doi: 10.1080/15384101.2025.2563930 (PMC12533958; doi:10.1080/15384101.2025.2563930)
Supplement: Supplemental Material [file KCCY_A_2563930_SM6482.docx]

Supplementary figures

**
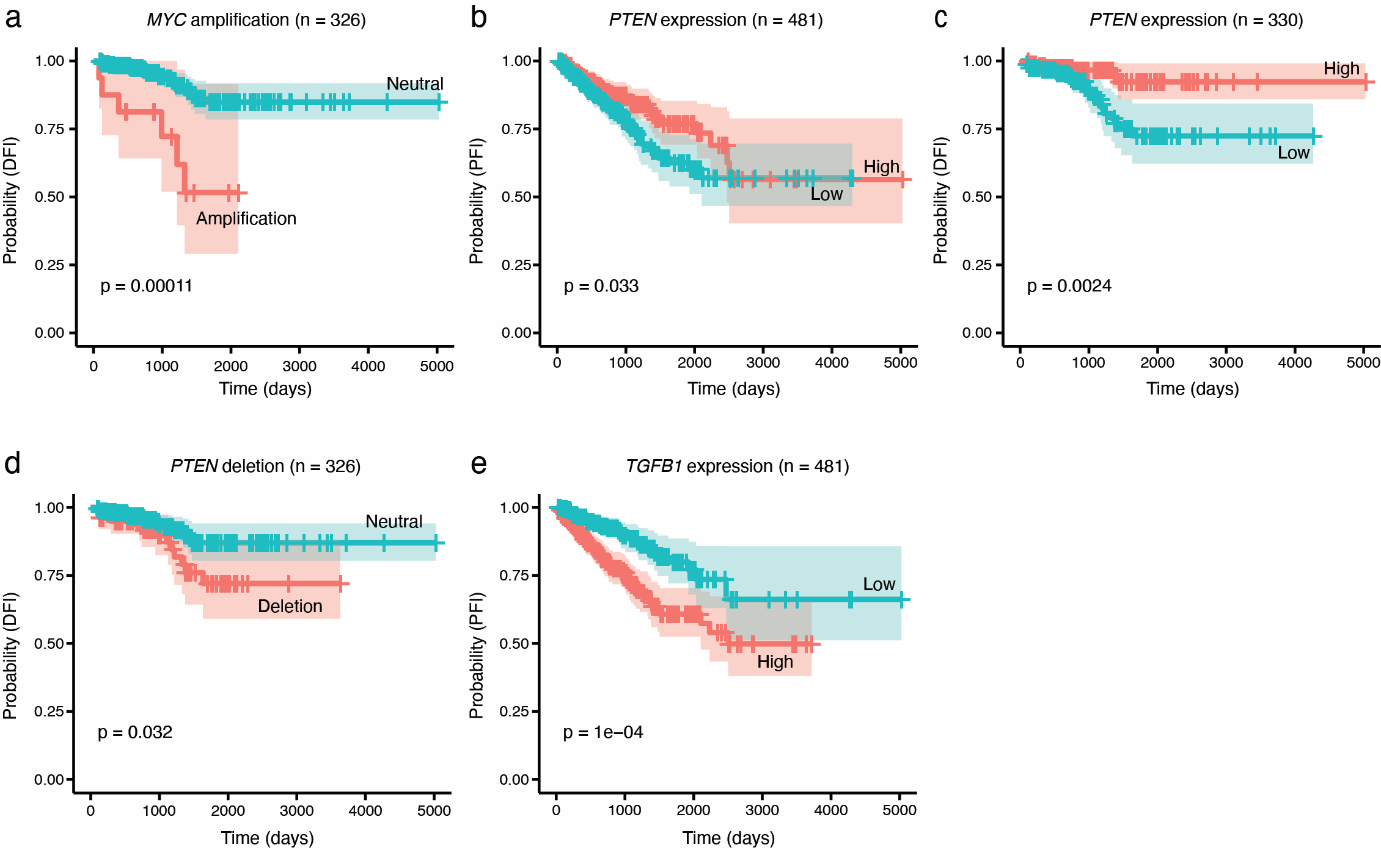
**

**Supplementary Figure S1.** Kaplan-Meier curves for significant molecular features. (a) Kaplan-Meier curves for *MYC* amplification for disease-free interval (DFI). (b) Kaplan-Meier curves for *PTEN* expression for progression-free interval (PFI). (c) Kaplan-Meier curves for *PTEN* expression for DFI. (d) Kaplan-Meier curves for *PTEN* deletion for DFI. (e) Kaplan-Meier curves for *TGFB1* expression for PFI.

**
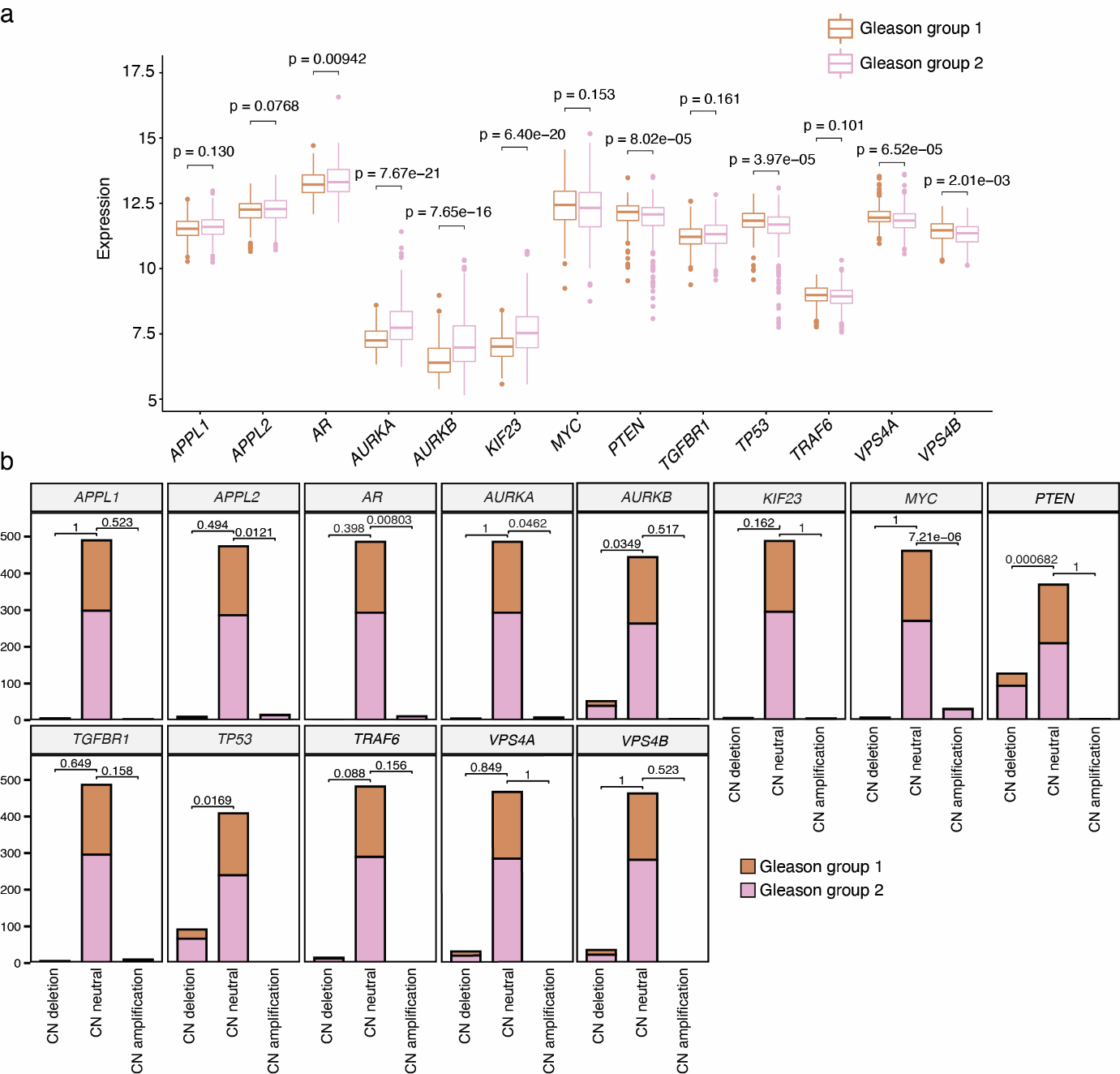
**

**Supplementary Figure S2.** Association between molecular features and Gleason group. (a) Box plots showing expression of the 13 genes of interest for the two Gleason groups. The p-values shown were obtained by comparing the expressions between Gleason groups using the Wilcoxon test. (b) Bar plots showing the distribution of Gleason groups with different copy number (CN) status. The p-values shown were obtained by comparing the proportion of individuals with CN amplification/deletion versus those with copy-neutral status using Fisher’s exact test. Gleason group 1: GS ≤ 6 or GS = 3 + 4; Gleason group 2: GS = 4 + 3 or GS ≥ 8.

**
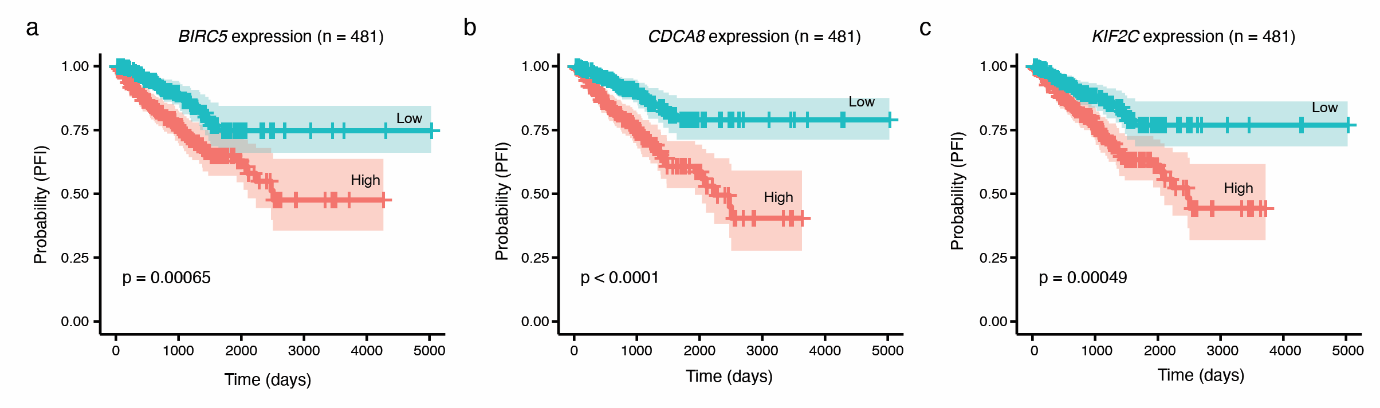
**

**Supplementary Figure S3.** Results from the univariate survival analysis in PRAD for additional genes. Kaplan-Meier curves illustrate the PFI of low- and high-expression groups of *BIRC5* (a), *CDCA8* (b), and *KIF2C* (c).


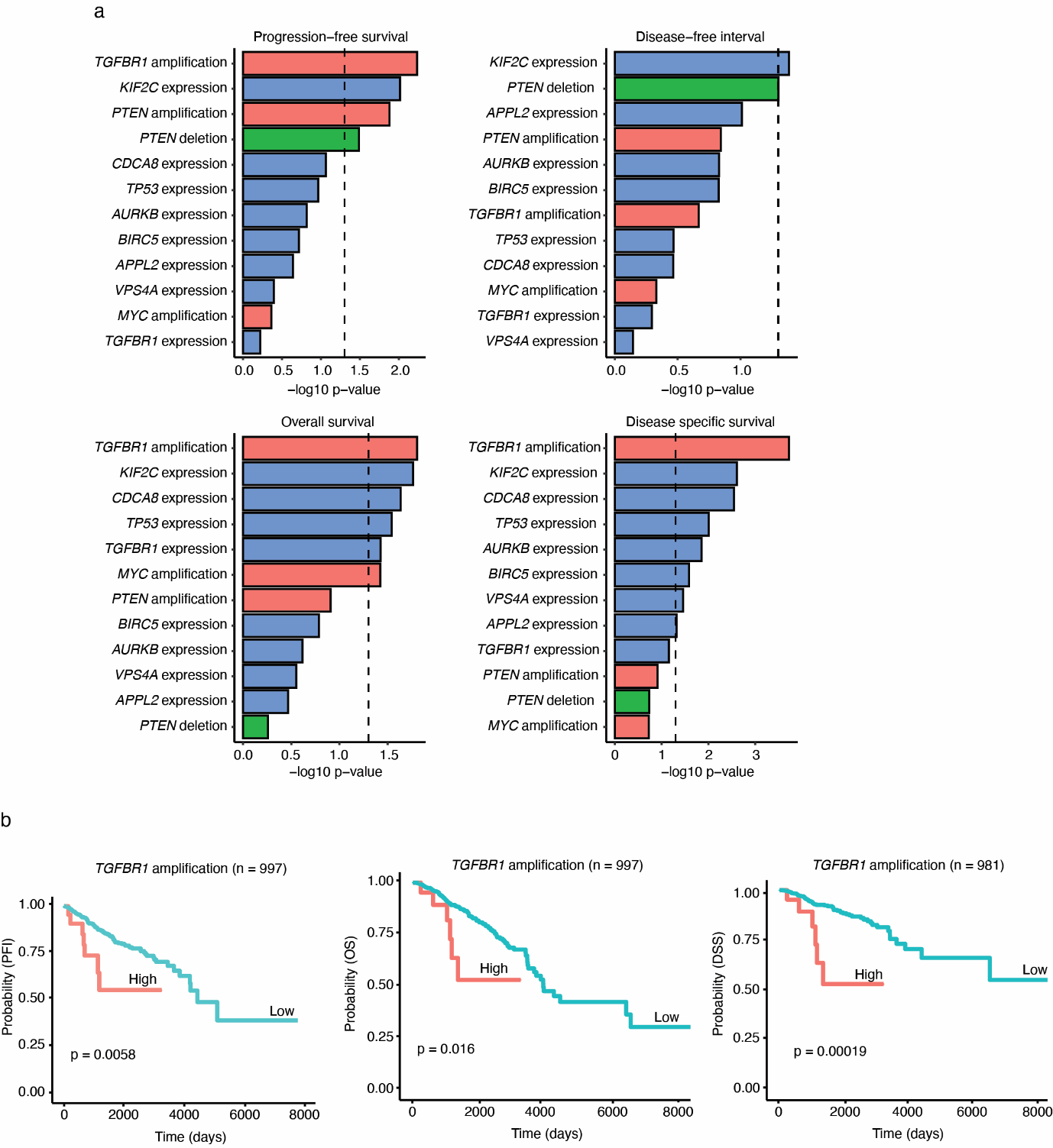


**Supplementary Figure S4.** Results from the univariate survival analysis in BRCA. (a) Survival analysis log-rank test results. Bar plot shows the −log10 p-values obtained for molecular features, based on univariate analysis. Dashed line indicates the threshold for significant features. (b) Kaplan-Meier curves for *TGFBR1* amplification for PFI, OS and DSS based on univariate analysis in the BRCA cohort.


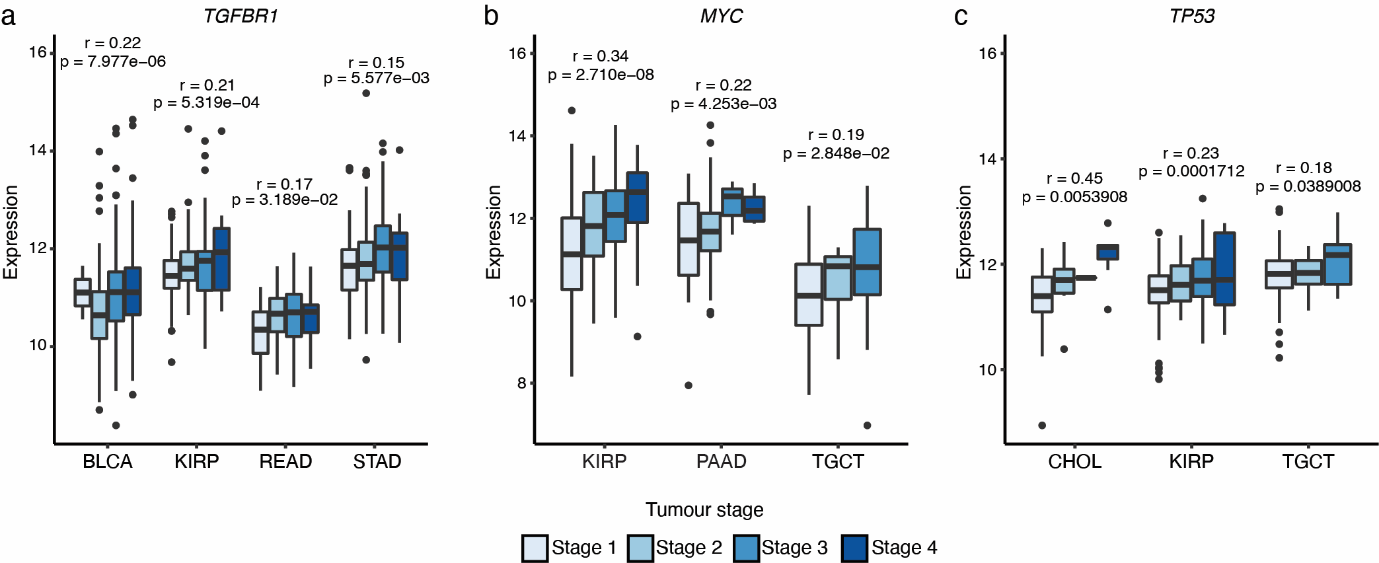


**Supplementary Figure S5.** Box plots showing the relationship between tumour stages and gene expression of *TGFBR1*, *MYC,* and *TP53* in different cancer types. (a) Box plots showing the distribution of expression of *TGFBR1* in bladder urothelial adenocarcinoma (BLCA), kidney renal papillary carcinoma (KIRP), rectum adenocarcinoma (READ), and stomach adenocarcinoma (STAD) for the four groups corresponding to the four cancer stages. (b) Box plots showing the distribution of expression of *MYC* in KIRP, pancreatic adenocarcinoma (PAAD), and testicular germ cell tumours (TGCT) for the four groups corresponding to the four cancer stages. (c) Box plots showing the distribution of expression of *TP53* in cholangiocarcinoma (CHOL), KIRP and TGCT for the four groups corresponding to the four cancer stages.
